# Inhibition of T cell-mediated inflammation in uveitis by a novel anti-CD3 antibody

**DOI:** 10.1186/s13075-017-1379-9

**Published:** 2017-07-25

**Authors:** Sunao Sugita, Jun Shimizu, Kenichi Makabe, Hiroshi Keino, Takeshi Watanabe, Masayo Takahashi

**Affiliations:** 1 Laboratory for Retinal Regeneration, Center for Developmental Biology, RIKEN, 2-2-3 Minatojima-minamimachi, Chuo-ku, Kobe, 650-0047 Japan; 2 Center for Innovation in Immunoregulative Technology and Therapeutics, Graduate School of Medicine, Kyoto University, Kyoto, Japan; 3 Department of Ophthalmology, Kyorin University School of Medicine, Tokyo, Japan; 4 The Tazuke-Kofukai Medical Research Institute and Kitano Hospital, Osaka, Japan

**Keywords:** Anti-CD3 antibody, T cells, Uveitis, Inhibition

## Abstract

**Background:**

A novel anti-mouse CD3ε antibody, Dow2, recognizes mouse CD3ε without activating T cells and suppresses T-cell activation. The purpose of this study was to determine whether Dow2 can inhibit T cells in uveitis.

**Methods:**

Experimental autoimmune uveitis (EAU) was induced in mice by immunization with retinal peptides, followed by administration of Dow2. Inflammation was evaluated by color fundus photography, optical coherence tomography, fluorescein angiography, and histology. Intraocular cells from EAU mice were used to examine the effect of Dow2 on retinal antigen-specific T cells. The effects of Dow2, conventional CD3ε antibodies, and isotype control immunoglobulin G (IgG) on splenic T cells were compared by assessing cell proliferation by the mixed lymphocyte reaction assay, inflammatory cytokine production by enzyme-linked immunosorbent assay (ELISA) and immunohistochemistry, and gene expression by quantitative reverse-transcription polymerase chain reaction (RT-PCR). T-cell subpopulations were characterized by flow cytometry to evaluate the expression of CD4, CD8, CD44, CD62L, and Foxp3.

**Results:**

Dow2 significantly reduced T-cell activation and counteracted activation associated with anti-CD3ε antibodies. Unlike conventional CD3ε antibodies, Dow2 treatment did not upregulate T helper (Th)1-/Th17-associated gene expression and cytokine production in splenic T cells. Interferon (IFN)-γ production by retinal antigen-specific T cells was also significantly reduced. Ocular inflammation was significantly reduced in Dow2-treated EAU mice compared to control EAU mice, with fewer T cells infiltrating into the retinas of Dow2-treated EAU mice. In immunohistochemistry, Th1 and Th17 cells invaded the retina in control EAU mice but not Dow2-treated EAU mice. No effects on peripheral T-cell numbers were observed following systemic administration of Dow2.

**Conclusion:**

The novel anti-CD3 antibody Dow2 can inhibit T cell-mediated inflammation in uveitis models. Thus, inhibition of T-cell activation by anti-CD3 therapy with this new antibody may protect uveitis patients from severe ocular inflammation.

## Background

Under severe inflammatory conditions, immune privilege in the eye may degrade and permit the infiltration of proinflammatory immune cells. These immune cells include T cells, B cells, macrophages/monocytes, microglia, neutrophils, natural killer (NK)/NKT cells, and dendritic cells, which may invade the retina, choroid, vitreous, and anterior chambers of the eye. Thus, intraocular inflammatory cells—especially T cells—play a significant role in the immune response involved in ocular inflammatory disorders.

Experimental autoimmune uveitis (EAU) models have been developed to investigate severe ocular inflammation. EAU models represent a T cell-mediated and T cell-specific form of autoimmune disease characterized by the infiltration of T cells and other immune cells into the retina [1–7]. Initiation of the major pathological events in EAU mouse models occurs through immunization with retinal antigens, which activates retinal antigen-specific T cells [7]. Over time, these activated T cells produce inflammatory cytokines, particularly T helper (Th)1 cytokines such as interferon (IFN)-γ and interleukin (IL)-2, which recruit inflammatory cells such as B cells, macrophages, and retinal microglia that can then cause retinal tissue damage. In addition, Th17 cells are also involved in ocular inflammation in human uveitis [8, 9] and EAU models [10, 11]. Thus, activated T cells are present in the retina as intraocular inflammation develops.

OKT3, an anti-human CD3 antibody (Ab), was the first monoclonal Ab to be used clinically [12–14]. OKT3 binds and interacts with the T-cell receptor (TCR) on T cells, temporarily activating and eventually inactivating T cells through anergy or apoptosis. However, within 24 h of treatment with OKT3, patients often experience an inflammatory cytokine storm involving such proinflammatory cytokines as IFN-γ, tumor necrosis factor (TNF)-α, and IL-2 as a consequence of initial T-cell activation [15, 16].

We recently established two novel anti-CD3 monoclonal Abs, anti-mouse Dow2 and anti-human 20-2b2, which downregulate TCR/CD3 in T cells [17, 18]. Dow2 recognizes mouse CD3ε without activating T cells [17], while 20-2b2 binds and modulates TCR on human T cells [18]. In contrast to OKT3, T cells are minimally activated following treatment with these Abs, TCR expression is downregulated in T cells, and there is neither T-cell proliferation nor a cytokine storm. Despite these promising observations, there have been no reports on whether these Abs can inhibit localized inflammation in conditions such as ocular inflammation.

Therefore, the purpose of this study was to determine whether the novel anti-mouse CD3 Ab Dow2 could inhibit T cells in EAU models.

## Methods

### Mice

Splenocytes were obtained from adult C57BL/6JJcl and BALB/c mice (CLEA Japan, Inc.). We induced EAU in 6- to 8-week-old female C57BL/6JJcl mice. The care and maintenance of the mice conformed to the Association for Research in Vision and Ophthalmology’s Statement for the Use of Animals in Ophthalmic and Vision Research, as well as to the guidelines for animal experiments at the RIKEN Center for Developmental Biology.

### Induction of EAU and administration of Dow2

To induce EAU, mice were subcutaneously immunized in the neck region with an emulsion containing 200 μg of interphotoreceptor retinoid-binding protein peptide (IRBP_1–20_; Eurofins Genomics) and *Mycobacterium tuberculosis* strain H37Ra (Difco) in complete Freund’s adjuvant (Difco), and intraperitoneally injected with 100 ng of pertussis toxin (Sigma) as an additional adjuvant [6]. Seven days after immunization, EAU mice were intraperitoneally injected with 2 μg of Dow2 or rat immunoglobulin G (rat IgG2a, κ isotype control; BD).

Inflammation was evaluated by color fundus imaging, optical coherence tomography (OCT), fluorescein angiography (FA), and histology. Funduscopic and OCT examinations were conducted on days 7, 14, and 21 postimmunization, and histological and FA examinations were conducted on day 21. Clinical scores [4] and OCT scores [19] were calculated as previously described. Splenocytes were harvested from EAU mice for flow cytometry analyses or to evaluate IRBP-induced cytokine production by retinal antigen-specific T cells in vitro. The supernatants of cultured splenocytes from EAU mice immunized with or without IRBP peptides were collected and mouse IFN-γ levels quantified by enzyme-linked immunosorbent assay (ELISA; R&D Systems).

### Preparation of splenocytes and assessment of the mixed lymphocyte reaction (MLR)

Splenocytes from C57BL/6JJcl and BALB/c mice were pressed through a 100-μm cell strainer to produce a single-cell suspension. Allogeneic immune responses in splenocytes were assessed by the MLR assay, with cell proliferation quantified by measuring carboxyfluorescein succinimidyl ester (CFSE; Molecular Probes). In brief, CFSE-labeled C57BL/6JJcl splenocytes (2 × 10^6^ cells/well) and irradiated (20 Gy) BALB/c splenocytes (2 × 10^5^ cells/well) were cocultured in 24-well plates with 1 μg/ml of Dow2, a control anti-mouse CD3ε Ab (clone 17A2; BioLegend), or an isotype control (rat IgG). After 96-h incubation, CFSE-labeled splenocytes were washed and proliferation analyzed by flow cytometry.

### Preparation of purified T cells and measurements of cytokines produced by T cells

Mouse pan-T cells were isolated using a pan-T cell isolation kit (MACS systems, Miltenyi Biotec). More than 95% of these cells were determined to be CD3^+^ by flow cytometry. C57BL/6JJcl splenocytes (2 × 10^6^ cells/well) were cultured with 1 μg/ml of the previously mentioned Abs (Dow2, 17A2, or rat IgG) in 24-well plates for 48 h. After incubation, the supernatants were collected and mouse IFN-γ or mouse IL-17 levels quantified by ELISA (R&D Systems). The cells were harvested for analysis of gene expression by quantitative reverse-transcription polymerase chain reaction (qRT-PCR).

### qRT-PCR

Total RNA from splenocytes cultured for 48 h in the presence of Dow2 was extracted using an RNA isolation kit (Roche Diagnostics) and reverse transcribed to generate cDNA (Transcriptor First Strand cDNA Synthesis kit, Roche Diagnostics). For qPCR, cDNA was amplified with a LightCycler 480 system (Roche Diagnostics) using a qRT-PCR master mix (Roche Diagnostics), Universal Probe Library primers, and probes (Roche Diagnostics). The primers and probes were as follows: *T*-*bet*, forward primer, 5′-caaccagcaccagacagaga-3′, reverse primer, 5′-acaaacatcctgtaatggcttg-3′, probe #19; *IFN*-*γ*, forward primer, 5′-atctggaggaactggcaaaa-3′, reverse primer, 5′-ttcaagacttcaaagagtctgaggta-3′, probe #21; *IL*-*1α*, forward primer, 5′-ttggttaaatgacctgcaaca-3′, reverse primer, 5′-gagcgctcacgaacagttg-3′, probe #52; *IL*-*2*, forward primer, 5′-gctgttgatggacctacagga-3, reverse primer, 5′-ttcaattctgtggcctgctt-3′, probe #15; *IL*-*17*, forward primer, 5′-cagggagagcttcatctgtgt-3′, reverse primer, 5′-gctgagctttgagggatgat-3′, probe #74; *IL*-*10*, forward primer, 5′-cagagccacatgctcctaga-3′, reverse primer, 5′-tgtccagctggtcctttgtt-3′, probe #41; *Foxp3*, forward primer, 5′-agaagctgggagctatgcag-3′, reverse primer, 5′-gctacgatgcagcaagagc-3′, probe #20; *GAPDH*, forward primer, 5′-agcttgtcatcaacgggaag-3′, reverse primer, 5′-tttgatgttagtggggtctcg-3′, probe #9. The relative expression of each gene of interest was calculated from triplicate samples using the comparative threshold cycle number and normalized to the *GAPDH* internal control.

### Flow cytometry analysis

Mouse pan-T cells isolated from C57BL/6JJcl donors were incubated with 1 μg/ml of Dow2 or a control anti-mouse CD3 Ab (clone 17A2 or clone 145-2C11; BD Biosciences) at 4 °C for 30 min. After primary Ab incubation, cells were washed and incubated with a secondary Ab (Alexa Fluor 488-conjugated anti-rat or anti-hamster; Invitrogen-Life Technologies) at 4 °C for 30 min. Dow2-pretreated pan-T cells that were incubated with 145-2C11 were also incubated with the secondary Alexa Fluor 488 Ab.

The expression of CD4 and CD8 on splenocytes from normal control mice and Dow2- or rat IgG-treated EAU mice was assessed by flow cytometry. The expression of CD44, CD62L, and Foxp3 on splenocytes from EAU mice was also evaluated. After blocking mouse Fc receptors (cells were treated with anti-mouse CD16/CD32 Abs at 4 °C for 15 min; BD PharMingen), the cells were incubated with anti-mouse CD4 (BD Pharmingen), CD8 (BioLegend), CD44 (BioLegend), CD62L (BioLegend), or isotype control (rat IgG) at 4 °C for 30 min. For the evaluation of intracellular Foxp3 expression, harvested T cells were permeabilized and incubated with anti-mouse Foxp3 (eBioscience). For permeabilization, these cells were treated with an intracellular staining material (BD Cytofix/Cytoperm Kits; BD PharMingen) to detect Foxp3 molecules. For detection of Th1/Th17 cells in spleens, we collected splenocytes from EAU mice, Dow2-, or rat IgG-treated EAU mice, and stained these cells with anti-mouse CD4, IFN-γ (R&D Systems), or IL-17 Abs (R&D Systems) after permeabilization. PE-conjugated mouse IgG (R&D Systems) was used as the isotype control. Cells (1 × 10^6^) were stained for 30 min at room temperature in the dark. All samples were analyzed on a FACSCanto II flow cytometer (BD). Data were analyzed with FlowJo software (version 9.3.1).

### Immunohistochemistry

Eyes from EAU mice were collected 21 days after immunization, fixed, and embedded in paraffin (Sigma-Aldrich). A series of five sequential paraffin-embedded sections (10-μm/section) were collected with an auto-slide preparation system (Kurabo).

For immunohistochemistry staining, the sections were blocked with 5% goat serum in phosphate-buffered saline (PBS) for 1 h at room temperature, and then incubated with primary rabbit Abs against mouse CD3 (Abcam), mouse CD4 (Abcam/BD PharMingen), mouse IFN-γ (Novus Biologicals), and mouse IL-17 (Abcam) at 4 °C overnight. After washing three times with PBS/Tween 20, sections were incubated with secondary Abs (Alexa Fluor 546-conjugated anti-rabbit) for 1 h at room temperature and counterstained with DAPI (Thermo Fisher Scientific). Images were acquired with a confocal microscope (LSM700, Zeiss).

### Statistical evaluation

Each experiment was repeated at least twice with similar results. Parametric data were analyzed by Student’s *t* test. Nonparametric data were analyzed by the Mann-Whitney *U* test. Values were considered statistically significant at *P* < 0.05.

## Results

### Novel anti-mouse CD3 Ab Dow2 suppresses T-cell proliferation and activation and does not stimulate T cells in vitro

**Fig. 1:**
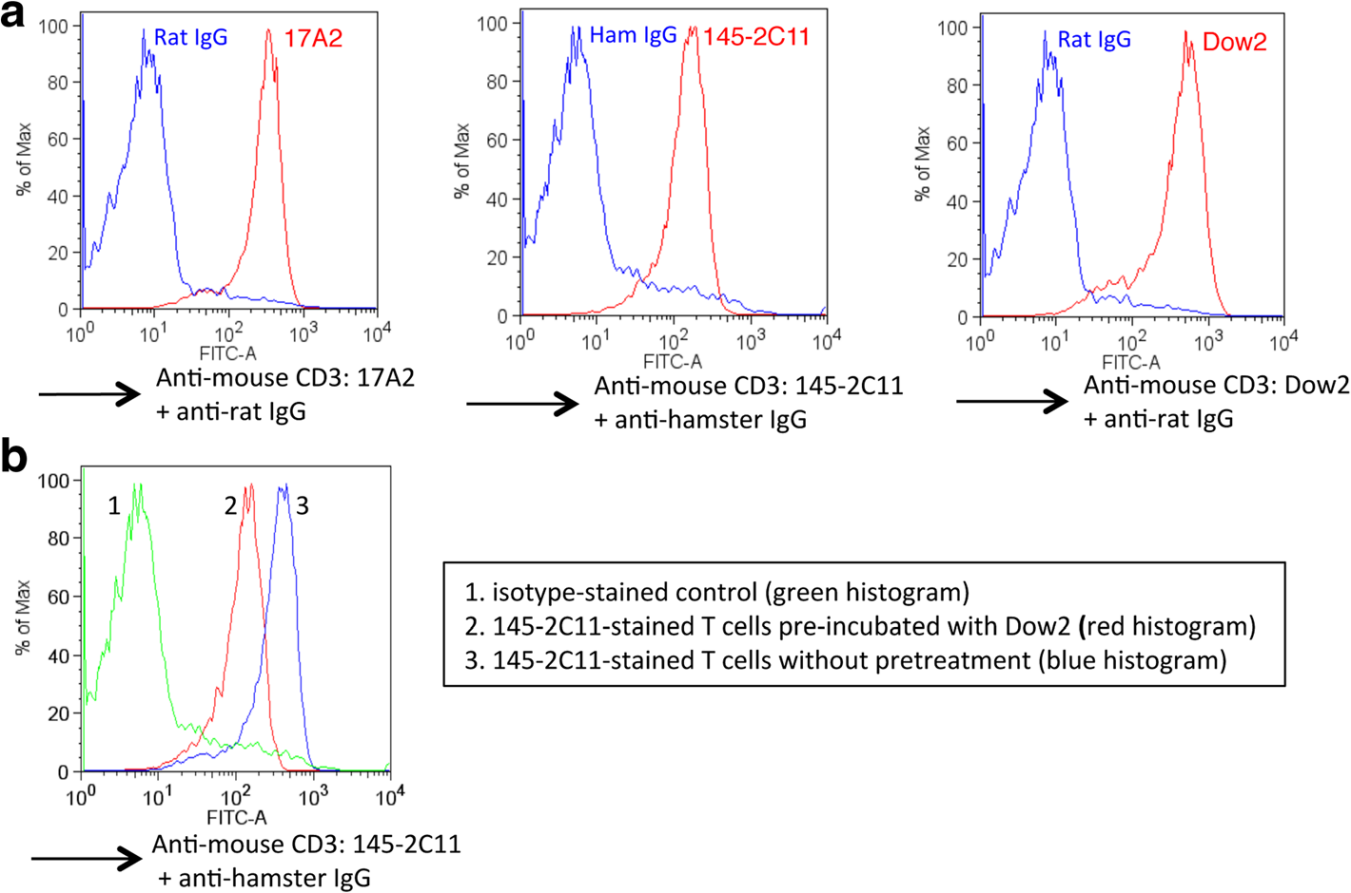
Recognition of CD3ε on mouse T cells by Dow2. **a** Suspensions of splenic pan-T cells from normal C57BL/6JJcl donors were used for flow cytometry analyses comparing the binding profiles of T cells incubated with Dow2 to T cells incubated with control anti-mouse CD3ε Abs 17A2 or 145-2C11 (1 μg/ml). In each panel, the *blue* histogram represents the isotype-stained control and the *red* histogram represents the T-cell population stained with one of the anti-mouse CD3 Abs. **b** Binding profile of pan-T cells preincubated with Dow2 for 2 h and subsequently stained with 145-2C11. Representative data from three independent FACS experiments with similar results are shown. *Ham IgG* hamster immunoglobulin G isotype control

Using flow cytometry, we first determined whether Dow2 can bind CD3 on mouse T cells by comparing its staining profile with two control anti-mouse CD3 Abs, 17A2 and 145-2C11. T cells stained with Dow2 demonstrated a clear separation from the control T-cell population, comparable to the separation observed for T cells stained with 17A2 or 145-2C11 (Fig. 1a). Interestingly, T cells preincubated with Dow2 had reduced binding with 145-2C11 (Fig. 1b), suggesting that Dow2 is similarly able to bind mouse CD3ε.

**Fig. 2:**
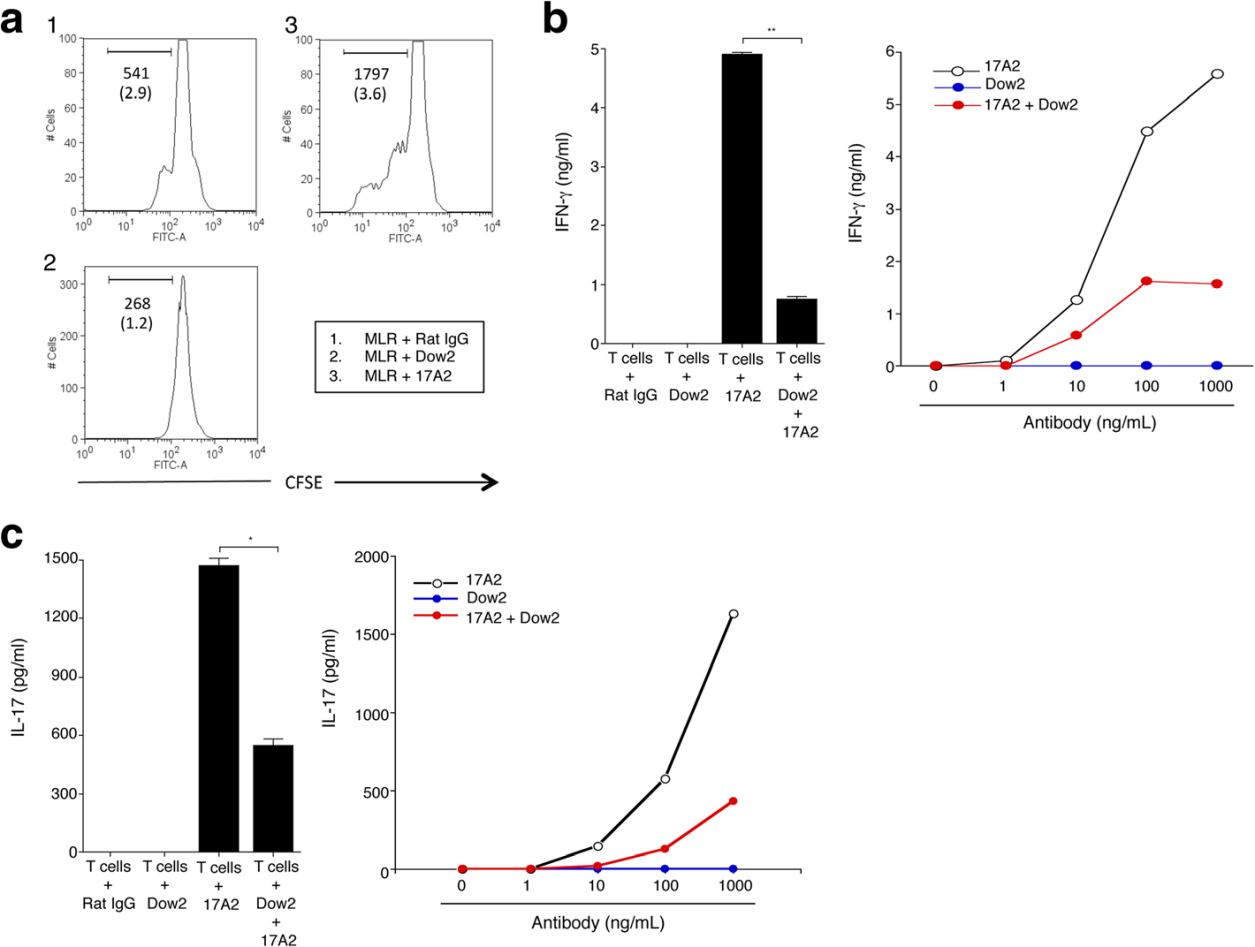
Suppression of T-cell activation by Dow2 in vitro. **a** T-cell proliferation was assessed following mixed lymphocyte reactions (*MLR*) involving carboxyfluorescein succinimidyl ester (*CFSE*)-labeled C57BL/6JJcl splenocytes cocultured with irradiated (20 Gy) BALB/c splenocytes in the presence of Dow2, 17A2, or isotype control (1 μg/ml). Each panel is annotated with the number of CFSE-positive cells and, in parentheses, the concentration of IFN-γ (ng/ml) in coculture supernatants as determined by ELISA. **b** *Left panel*: IFN-γ production by pan-T cells following incubation with Dow2, 17A2, Dow2 + 17A2, or rat IgG (1 μg/ml). **c** *Left panel*: IL-17 production by T cells following incubation with Dow2, 17A2, Dow2 + 17A2, or rat IgG. Results of triplicate samples are presented as the mean ± SEM. *Right panels*: IFN-γ (**b**) or IL-17 (**c**) production by pan-T cells following incubation with Dow2 (0–1000 ng/ml) and/or 17A2 (0–1000 ng/ml). Representative data from three independent experiments with similar results are shown. *P < 0.05, **P < 0.005

We next determined whether Dow2 can suppress lymphocyte proliferation in vitro with the MLR assay. CFSE-labeled C57BL/6JJcl splenocytes cocultured with irradiated BALB/c splenocytes were incubated with Dow2, 17A2, or isotype control. Compared with the control, 17A2 treatment greatly enhanced proliferation (Fig. 2a). In contrast, Dow2 did not promote a proliferative response.

We also examined whether Dow2 could suppress T-cell activation in vitro. Purified mouse T cells were incubated with Dow2, 17A2, both Dow2 and 17A2, or rat IgG isotype control. Incubation with Dow2 alone or rat IgG did not stimulate T cells to produce detectable IFN-γ (Fig. 2b, left panel) or IL-17 (Fig. 2c, left panel). In contrast, T cells incubated with 17A2 produced increased levels of cytokines which were significantly reduced in T cells incubated with both Dow2 and 17A2. T cells incubated with increasing concentrations of Dow2 (0–1000 ng/ml) consistently produced no detectable IFN-γ, whereas T cells incubated with 17A2 demonstrated a clear dose-response relationship between 17A2 (0–1000 ng/ml) and production of IFN-γ/IL-17 (Fig. 2b and c, right panel). However, T cells incubated with both Abs produced less IFN-γ/IL-17 compared to T cells incubated with 17A2 only. These results suggest that Dow2 could suppress T cell activation in vitro.

**Fig. 3:**
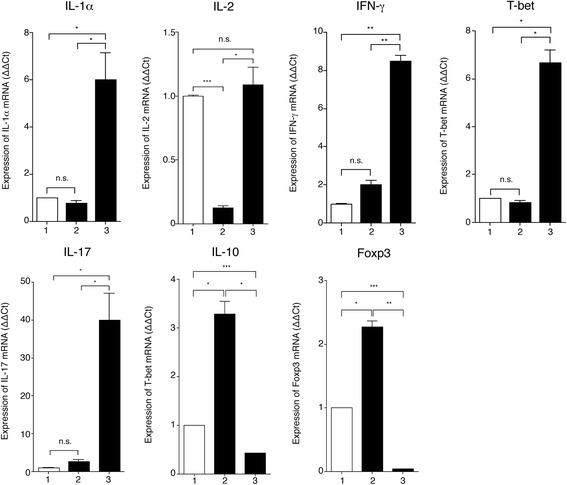
Suppression of inflammatory cytokine gene expression in splenic T cells by Dow2. Quantification of mRNA levels for *IL*-*1α*, *IL*-*2*, *IFN*-*γ*, *T*-*bet*, *IL*-*17*, *IL*-*10*, and *Foxp3* in mouse splenocytes incubated with Dow2, 17A2, or rat IgG isotype control. Total RNA was extracted from cells, reverse transcribed, and amplified by qRT-PCR with primers for these genes and *GAPDH*. Results present the relative expression of each gene compared to cells incubated with rat IgG (**ΔΔ**Ct; splenocytes + rat IgG = 1). Representative data from four independent experiments with similar results are shown. Each bar represents the mean ± SEM. *P < 0.05, **P < 0.005, ***P < 0.0005. *1* splenocytes + rat IgG isotype control, *2* splenocytes + Dow2, *3* splenocytes + 17A2, *n.s*. not significant

To further characterize Dow2, we next examined whether Dow2 can suppress the expression of genes associated with T-cell activation, particularly regulatory T cell (Treg)-, Th1-, or Th17-associated genes. To quantify the expression of *IL*-*1α*, *IL*-*2*, *IFN*-*γ*, *T*-*bet*, *IL*-*17*, *IL*-*10*, and *Foxp3*, we performed qRT-PCR on total RNA extracted from mouse splenocytes incubated with Dow2, 17A2, or rat IgG. As expected, cells incubated with Dow2 did not have increased mRNA levels for these Th1 genes; furthermore, cells incubated with Dow2 had significantly reduced levels of *IL*-*2* compared with controls (Fig. 3) (*P* < 0.0005). In contrast, cells incubated with 17A2 had significantly elevated mRNA levels for these Th1 genes, with the exception of *IL*-*2*, compared with controls. We obtained similar results for IL-17 (Th17-associated cytokine). Moreover, cells incubated with Dow2, but not 17A2, had significantly elevated mRNA levels for Treg-associated genes such as *IL*-*10* and *Foxp3* (*P* < 0.05). These results indicate that Dow2 could suppress the Th1/Th17 response in splenocytes including T cells at the level of gene expression. In addition, T cells treated with Dow2 may acquire Treg phenotype.

### Dow2 can suppress T cell activation in vivo in an EAU model

**Fig. 4:**
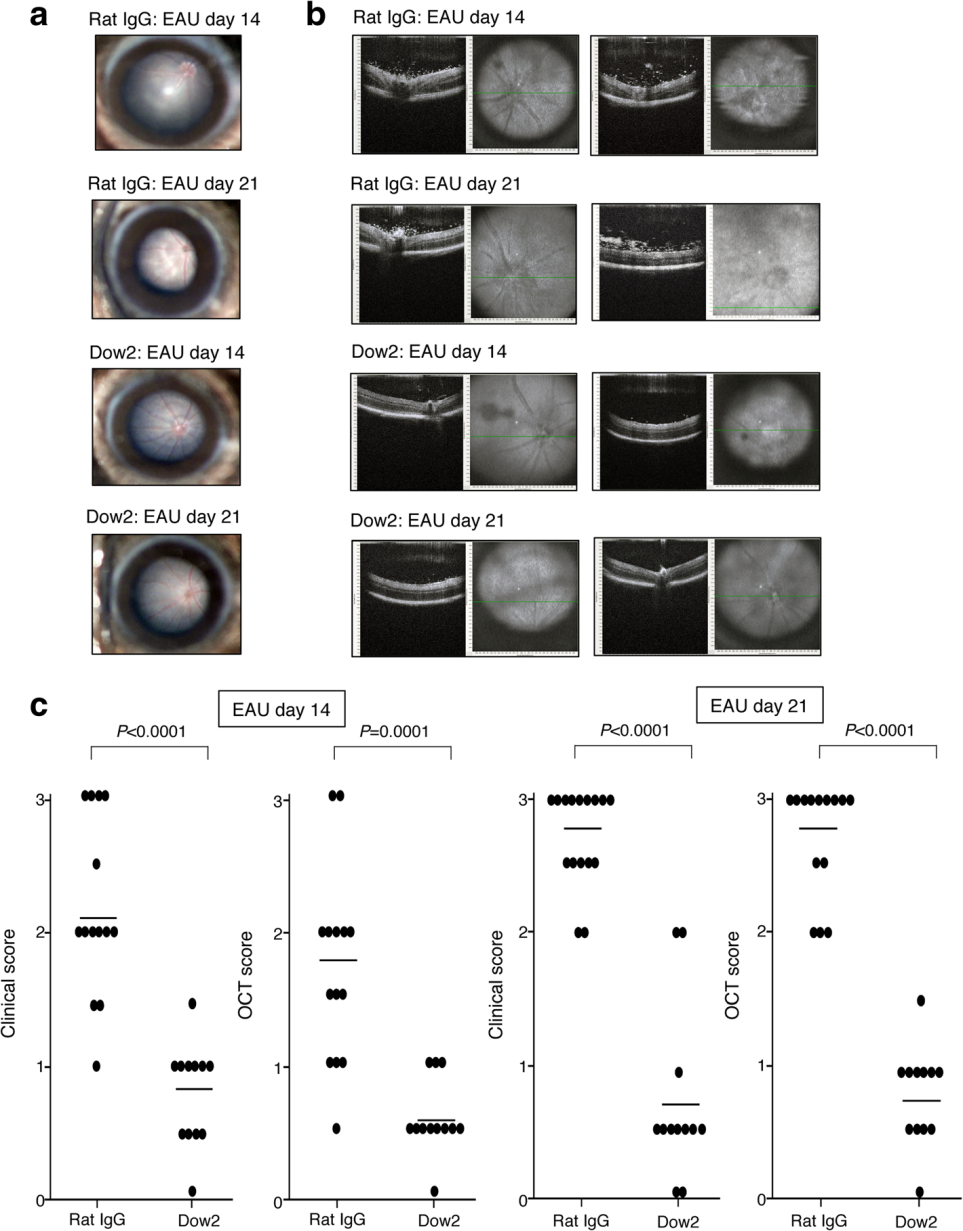
Suppression of ocular inflammation in vivo by Dow2 in experimental autoimmune uveitis (*EAU*) mice. Ocular inflammation was evaluated by (**a**) color fundus imaging, (**b**) optical coherence tomography (*OCT*), and (**c**) clinical and OCT scores in EAU mice treated at 7 days postimmunization with Dow2 or rat IgG isotype control (2 μg/mouse intraperitoneally). Clinical scores (fundus score, grades 0–4) and OCT scores (grades 0–4) were determined on days 14 and 21 postimmunization. *Filled ovals* represent individual scores, and *horizontal b*ars indicate the mean score for each treatment group (Dow2 mice, *n* = 12; control mice, *n* = 13). Representative data from five independent experiments with similar results are shown

To evaluate the effect of Dow2 in vivo, we investigated whether treatment with Dow2, compared to treatment with rat IgG isotype control, could suppress ocular inflammation in a mouse model of EAU. In EAU, ocular inflammation is associated with a T cell-dependent (especially Th1-type) immune response [2, 3, 5]. In rat IgG-treated control EAU mice, we detected pronounced ocular inflammation characterized by retinal exudates observable by color fundus imaging, along with retinal nodules and vitreous cell infiltration observable by OCT (Fig. 4a and b). In contrast, there was minimal to no ocular inflammation detected in Dow2-treated mice. Similarly, treatment with Dow2 significantly reduced ocular inflammation assessed by clinical and OCT scoring on days 14 and 21 postimmunization (Fig. 4c).

**Fig. 5:**
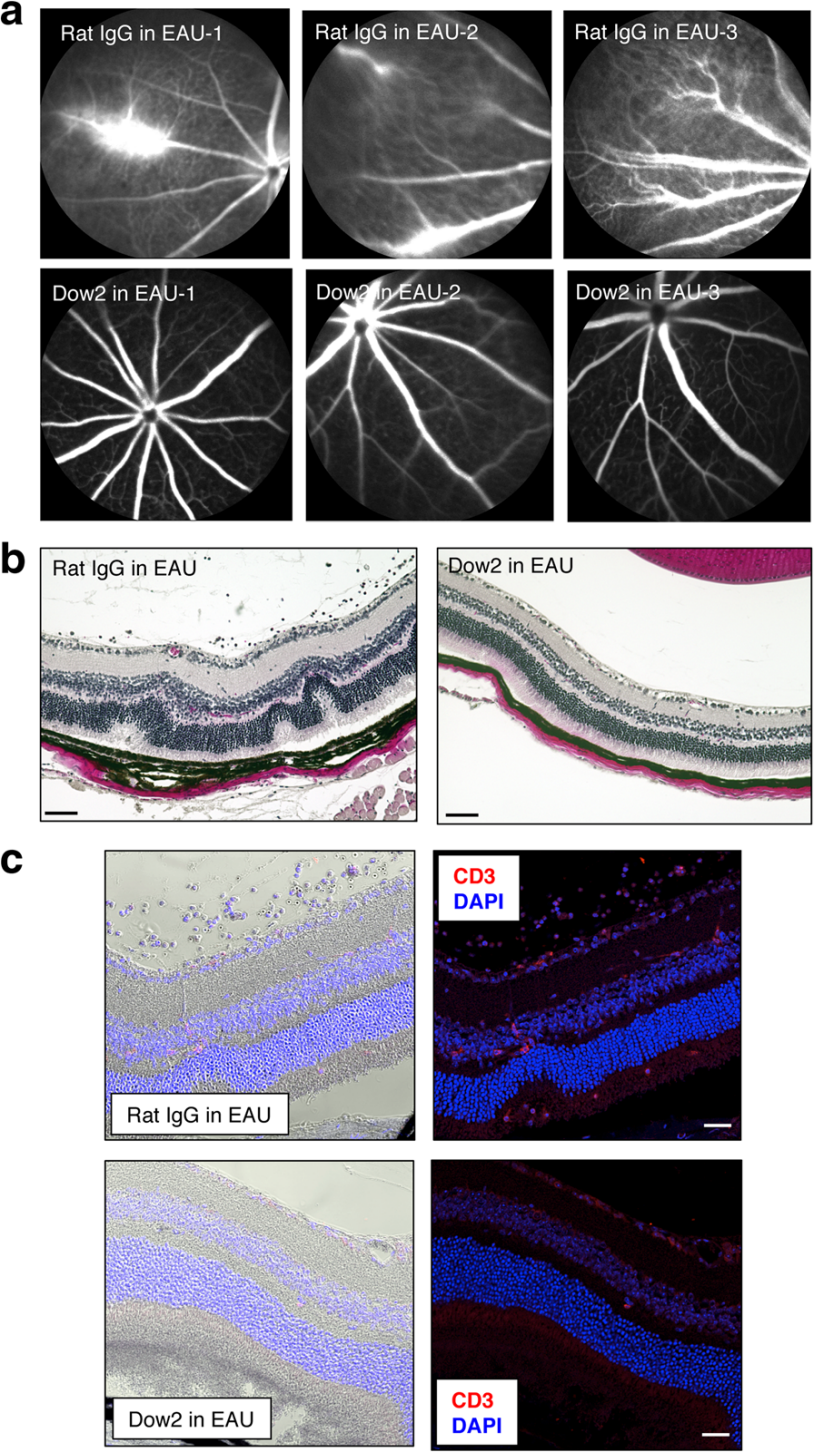
Evaluation of ocular inflammation in experimental autoimmune uveitis (*EAU*) mice treated with Dow2 or rat IgG isotype control. At 21 days postimmunization, the retinas of EAU mice were examined by FA, hematoxylin and eosin (H&E) staining of paraffin-embedded sections, and immunohistochemistry for CD3^+^ cells. Representative images are shown for (**a**) FA, (**b**) H&E staining of retinal sections (*scale bar* = 20 μm), and (**c**) immunohistochemistry of retinal sections (*scale bar* = 20 μm). *Left panels* present bright field images and *right panels* present fluorescence images stained for CD3 (*red*) and with DAPI (*blue*). For FA, representative data from three independent experiments with similar results are shown. For H&E staining and immunohistochemistry, *n* = 8 eyes per group

When FA was used to study the retinal microvasculature of EAU mice, we observed that Dow2-treated mice had retinal vessels similar to those of healthy mice, whereas retinal vessels of control mice exhibited severe inflammation (Fig. 5a). Consistent with this observation, hematoxylin and eosin (H&E)-stained retinal sections from control mice exhibited signs of inflammation together with inflammatory cell infiltration (Fig. 5b). In contrast, no retinal inflammation was observed in sections from Dow2-treated EAU mice. When retinal sections from both treatment groups were examined by immunohistochemical staining for CD3, few CD3^+^ T cells were observed in the retinas of Dow2-treated mice, whereas numerous CD3^+^ cells were observed in the retinas and vitreous spaces of control mice (Fig. 5c).

**Fig. 6:**
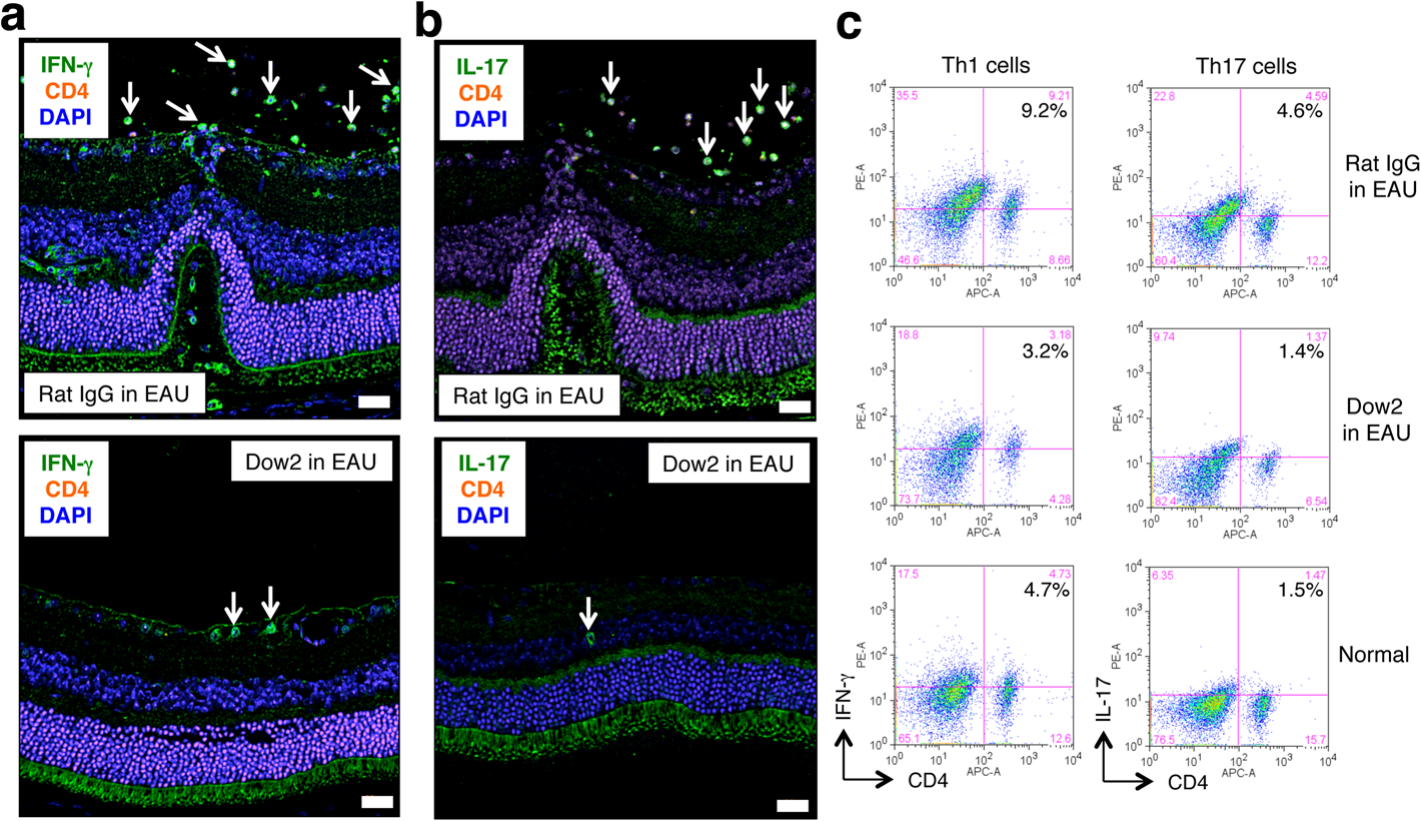
Suppression of Th1 and Th17 cells in Dow2-treated uveitis models. Representative images are shown for immunohistochemistry of retinal sections in experimental autoimmune uveitis (*EAU*) (Dow2 or rat IgG isotype control). Fluorescence images stained for (**a**) IFN-γ (*green*), CD4 (*red*), and with DAPI (*blue*), and (**b**) IL-17 (*green*), CD4 (*red*), and with DAPI (*blue*); *n* = 3 eyes per group. *Scale bar* = 20 μm. (**c**) Splenocytes were collected from Dow2- or rat IgG-treated EAU mice to detect Th1/Th17 cells after administration of these Abs. Percentages indicate double-positive CD4^+^IFN-γ^+^ or CD4^+^IL-17^+^ cells (*n* = 3 per group)

When we observed Th1/Th17 cells in Dow2-treated EAU mice, similar results were obtained in vitro. For example, numerous CD4^+^IFN-γ^+^ cells (i.e., Th1 cells) and CD4^+^IL-17^+^ cells (i.e., Th17 cells) were found in the retinas and vitreous spaces of control mice (Fig. 6a and b). In contrast, minimal Th1/Th17 cells were seen in the retinas of Dow2-treated mice (Fig. 6a and b). We also examined whether Th1/Th17 cells were included among splenocytes from Dow2-treated EAU mice. Compared with control EAU mice, CD4^+^IFN-γ^+^ Th1 and CD4^+^IL-17^+^ Th17 cell populations were decreased among splenocytes from Dow2-treated EAU mice (Fig. 6c). These results indicate that Dow2 inhibits the activation of infiltrated T cells and/or inhibits infiltration of T cells in vivo in a model of ocular inflammation.

### Dow2 also suppresses retinal antigen-specific T cells in vitro

**Fig. 7:**
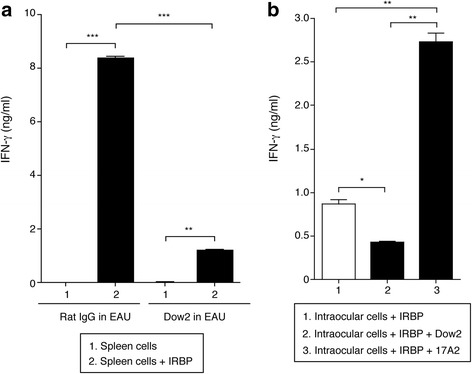
Capacity of Dow2 to suppress retinal antigen-specific T cell activation in vitro. IFN-γ production was measured by ELISA in culture supernatants of (**a**) splenocytes isolated from Dow2- or rat IgG-treated experimental autoimmune uveitis (*EAU*) mice (day 21 after immunization; *n* = 3 mice per group), cultured with or without Interphotoreceptor retinoid-binding protein (*IRBP*) retinal peptides for 48 h; and (**b**) intraocular cells from EAU mice with ocular inflammation (*n* = 12) cultured with IRBP retinal antigens and Dow2 or 17A2 for 48 h. Results of triplicates are presented as the mean ± SEM. *P < 0.05, **P < 0.005, ***P < 0.0005

We next examined whether Dow2 can suppress retinal antigen-specific T cells in vitro by collecting splenocytes from EAU mice treated with rat IgG isotype control or Dow2 and then culturing the cells with or without IRBP retinal peptides. When cultured with retinal antigens, splenocytes from control mice secreted significantly higher levels of IFN-γ compared to cells cultured without retinal antigens (Fig. 7a) (*P* < 0.0005). Compared to splenocytes from control mice, splenocytes from Dow2-treated mice secreted less IFN-γ when cultured with retinal antigens.

Intraocular cells collected from EAU mice that were untreated with Abs also produced IFN-γ when cultured with IRBP retinal antigens (Fig. 7b). The IRBP-stimulated production of IFN-γ was significantly reduced when EAU ocular cells were incubated with Dow2 (*P* < 0.05). In contrast, IRBP-stimulated production of IFN-γ was significantly enhanced in ocular cells incubated with 17A2 (*P* < 0.005). These results indicate that, unlike a conventional anti-mouse CD3 antibody, Dow2 can suppress the activation of retinal antigen-specific T cells such as primed T cells.

### Effect of Dow2 administration on peripheral T cells in EAU mice

**Fig. 8:**
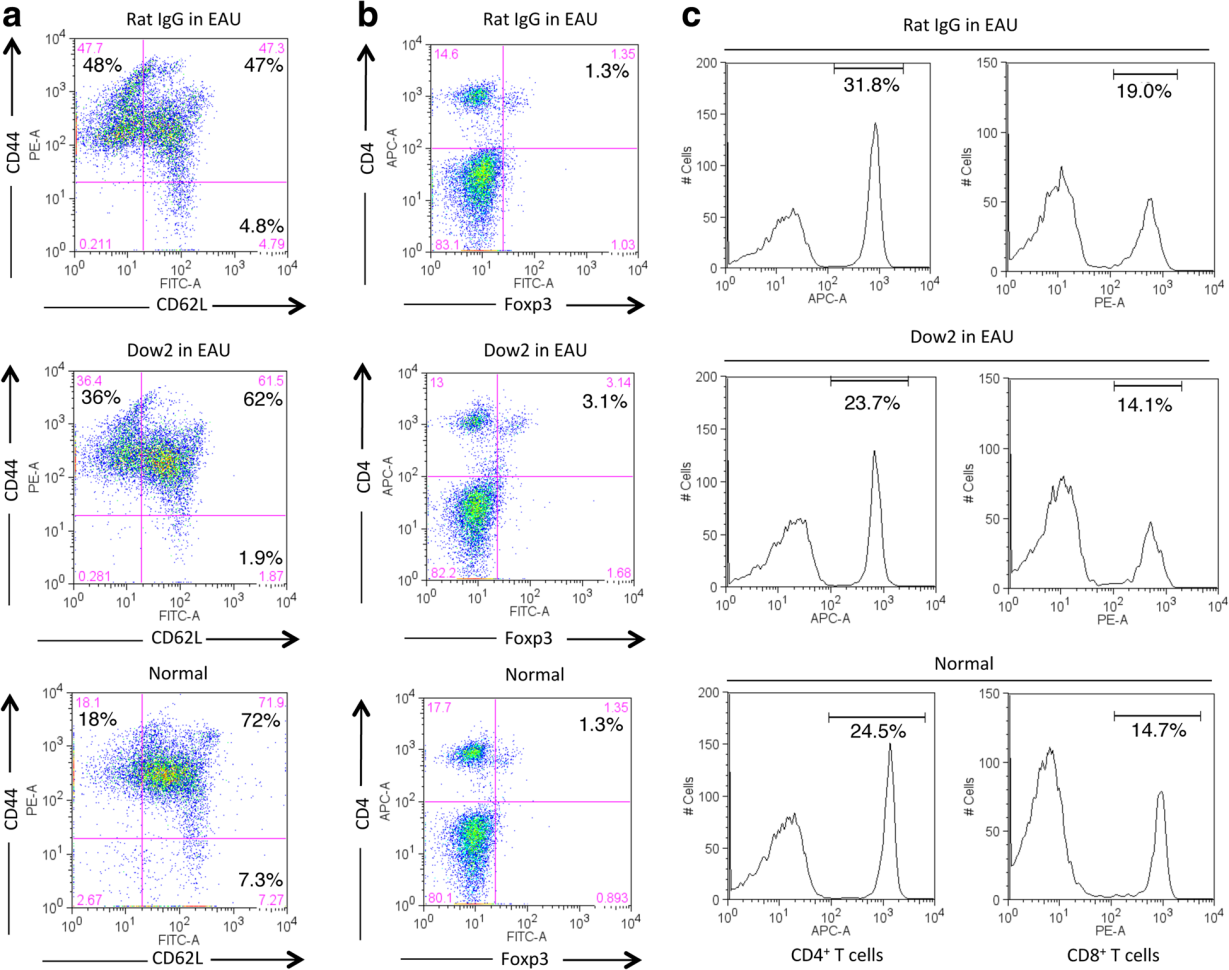
Effect of systemic Dow2 administration on peripheral T cells in experimental autoimmune uveitis (*EAU*) mice. Subpopulations of peripheral T cells were characterized by flow cytometry following splenocyte collection from normal mice, Dow2-treated EAU mice, and rat IgG-treated EAU mice. Splenocytes were analyzed for expression of (**a**) CD44/CD62L, (**b**) CD4/Foxp3, and (**c**) CD4 and CD8. In (**c**), percentages indicate the proportion of CD4^+^ or CD8^+^ cells. Representative data from four independent FACS experiments with similar results are shown

To gain further understanding of the systemic effects of Dow2, we examined peripheral T cells collected from the spleens of EAU mice treated with Dow2 or rat IgG isotype control. First, we examined the ratio of CD44^+^ effector memory T cells to CD62L^+^ naive T cells. Compared with normal mice, the ratio of memory T cells to naive T cells increased in control EAU mice (Fig. 8a). Compared with control EAU mice, both memory and naive T cells were decreased in Dow2-treated EAU mice. Interestingly, the number of CD4^+^Foxp3^+^ T cells was increased in Dow2-treated EAU mice compared with normal or control EAU mice (Fig. 8b).

Finally, we quantified the number of CD4^+^ and CD8^+^ splenic T cells. We observed similar numbers of these splenic T-cell populations for normal and Dow2-treated EAU mice in contrast to greater numbers of CD4^+^ and CD8^+^ T cells for control EAU mice (Fig. 8c). Thus, we observed no effect of systemic administration of Dow2 on peripheral T-cell numbers.

Taken together, our results indicate that Dow2 can bind CD3 on effector T cells and effectively suppress T-cell activation in inflammatory conditions.

## Discussion

In this study, we showed that treatment with a novel rat IgG_2a_ anti-mouse CD3ε Ab, Dow2, significantly reduced ocular inflammation in EAU models of noninfectious human uveitis. Previously, it was not well understood whether an anti-CD3 Ab could affect inflammation in the eye. Using an in vivo EAU model, we demonstrated that Dow2 could inhibit T cell-mediated ocular inflammation. Using in vitro models, we observed that Dow2 greatly suppressed T-cell activation, as indicated by the MLR assay. Importantly, Dow2 does not stimulate T cells. In contrast to conventional anti-mouse CD3ε antibodies, Dow2 downregulated the expression of Th1-/Th17-associated cytokine genes (*IFN*-*γ*, *IL*-*2*, *T*-*bet*, *IL*-*17*, and *IL*-*1α*) in mouse splenic T cells. In contrast, Dow2 upregulated the expression of Treg-associated cytokine genes such as *IL*-*10* and *Foxp3*. Furthermore, the production of inflammatory cytokines (IFN-γ/IL-17) by retinal antigen-specific T cells was reduced in Dow2-treated cells compared with controls. More importantly, we observed no effects in vivo on peripheral T-cell numbers after systemic administration of Dow2, suggesting that Dow2 treatment would not deplete T cells. In addition, when we examined the ratio of effector memory T cells (CD44^+^CD62L^–^) to naive T cells (CD44^–^CD62L^+^), the ratio of memory T cells to naive T cells increased in control EAU mice. Conversely, compared with control EAU mice, both memory and naive T cells were decreased in Dow2-treated EAU mice, indicating that Dow2 alters specific subpopulations of peripheral T cells. Because the number of memory effector T cells was decreased in Dow2-treated EAU mice, such T cells would be unlikely to infiltrate the eye. Furthermore, the relative decrease in naive T cells in Dow2-treated EAU mice compared with that in normal mice may indicate that Dow2 can bind to T cells and counteract stimulation.

Recently, we established that the anti-mouse CD3ε Ab Dow2 functionally downregulates the expression of TCR/CD3 on murine T cells [17]. Unlike conventional anti-mouse CD3ε Abs (e.g., 145–2C11), Dow2 does not activate T cells and can induce T-cell anergy. In our previous study, we found that administration of Dow2 in vivo effectively prolonged the survival of cardiac allografts [17]. In addition, target recognition or the determinant recognized by Dow2 is close to but differs from that recognized by anti-mouse CD3ε Abs 145–2C11. The immunosuppression induced by Dow2 is more effective than that induced by 145–2C11 in terms of delaying rejection in a mouse heart transplantation model, e.g., an intravenous injection of 145–2C11 to BALB/c mice strongly induced the release of IL-2. In contrast, these cytokine levels produced by Dow2-treated mice were markedly reduced. Furthermore, we observed that Dow2 appeared to upregulate Treg suppressive activity while having no effect on Treg induction [17]. As shown in our current study, the number of CD4^+^Foxp3^+^ T cells in Dow2-treated EAU mice was increased compared with normal or control EAU mice. These results suggest that Dow2 may provide a new means for reducing the inflammatory response and inducing immunosuppression by modulating Treg activity.

In contrast, T cells are temporarily activated and eventually inactivated following clinical treatment with the anti-human CD3 antibody OKT3 [12–14]. This T-cell activation increases the risk for an inflammatory cytokine storm and related adverse effects such as fever, muscular pain, and headache [15, 16]. In addition, some patients treated with OKT3 suffered from infections or cancer, possibly due to the depletion of T cells. Thus, while OKT3 was the first monoclonal Ab to be used clinically, modern use of OKT3 in transplantation medicine has decreased due to its adverse effects. Studies in vivo examining the 145-2C11 agonistic anti-mouse CD3ε antibody found that it induced IL-2 production and promoted T-cell proliferation. In contrast, much less IL-2 was secreted in vivo following treatment with Dow2 [17]. Consistent with this previous observation, we found that mouse splenocytes incubated with Dow2 had significantly reduced *IL*-*2* mRNA levels compared with rat IgG-treated control cells (Fig. 3), suggesting that Dow2 may further reduce production of inflammatory cytokines. Although the mechanisms by which T cells are suppressed during Dow2 treatment are still unknown, our previous study suggests that, concerning signaling mechanisms, LAT and PLCγ1 phosphorylation was significantly impaired in Dow2-induced anergic T cells [17].

Ke et al. previously reported that both systemic and local injection of anti-mouse CD3 monoclonal Abs in EAU mice greatly reduced ocular inflammation [20]. Similar to our results, they showed that anti-CD3 treatment increased the percentage of CD4^+^Foxp3^+^ Treg cells. Furthermore, when T cells collected from IRBP-immunized, anti-CD3-treated mice were stimulated with IRBP in the presence of antigen-presenting cells, supernatants from these T-cell cultures contained significantly higher levels of IL-10 and transforming growth factor (TGF)-β1 compared with control cultures [20], suggesting that anti-CD3 monoclonal Ab treatment ameliorates EAU by inducing Treg cells. In addition to CD3, other studies have investigated whether anti-mouse monoclonal Ab to cytokines and cytokine receptors can mitigate autoimmune uveitis. For instance, anti-TNF-α therapy was observed to suppress ocular inflammation in vivo [21] and in vitro [22], and anti-IL-6R therapy suppressed ocular inflammation in EAU models [23, 24]. Both anti-TNF-α and anti-IL-6R therapies can induce Treg cells and inhibit Th1-/Th17-type inflammatory T cells. Because these two therapies presumably act through different suppressive mechanisms, they represent potential avenues to increase treatment options for severe uveitis in patients. To further improve treatment options, we are now investigating whether a novel anti-human CD3 monoclonal antibody, 20-2b2 [18], can suppress ocular inflammation in an autoimmune uveitis monkey model [25].

Treating EAU as early as in this study does not represent the true condition of patients with uveitis, who have active retinitis at the time of therapy. Therefore whether Dow2 can suppress a well-established disease should be evaluated. However, as shown in this study, Dow2 likely suppresses the onset of disease because the timing of antibody injections was during the induction phase of immunization for EAU. To verify that Dow2 actually suppresses uveitis, we must first confirm all mice have uveitis, as scored by fundus examination, and then administer Dow2. For clinical application of Dow2, timing and route of administration must be determined for patients with uveitis.

## Conclusions

The novel anti-CD3 Ab Dow2 is able to suppress T-cell activation in vitro and in vivo. T cells treated with Dow2 failed to acquire effector T-cell functionality, as indicated by a lack of inflammatory Th1-/Th17-associated cytokine production. Importantly, administration of Dow2 greatly reduced ocular inflammation in a mouse model of autoimmune uveitis. Thus, suppression of effector T cells by anti-CD3 therapy may protect uveitis patients from severe ocular inflammation.

## Abbreviations

Ab: Antibody
CFSE: Carboxyfluorescein succinimidyl ester
EAU: Experimental autoimmune uveitis
ELISA: Enzyme-linked immunosorbent assay
FA: Fluorescein angiography
IFN: Interferon
IgG: Immunoglobulin G
IL: Interleukin
IRBP: Interphotoreceptor retinoid-binding protein
MLR: Mixed lymphocyte reaction
OCT: Optical coherence tomography
PBS: Phosphate-buffered saline
qRT-PCR: Quantitative reverse-transcription polymerase chain reaction
TCR: T-cell receptor
Th: T helper
TNF: Tumor necrosis factor
Treg: Regulatory T cell
